# Supplementary material for: Efficient and reliable spike sorting from neural recordings with UMAP-based unsupervised nonlinear dimensionality reduction
Source: PLoS Biol. 2025 Nov 24;23(11):e3003527. doi: 10.1371/journal.pbio.3003527 (PMC12671831; doi:10.1371/journal.pbio.3003527)
Supplement: S1 Fig — This panel shows how each approach performs on four electrodes (A–D) during a time interval comparison task, where different colors denote the spikes assigned to putative neurons. HDBSCAN stands out for two key reasons: (1) it can reveal clusters with unusual shapes and densities (see electrodes A–D), and (2) it avoids forcing uncertain spikes (shown in pink) into any cluster. These unassigned points typically represent noise or non-neuronal signals, and excluding them sharpens the overall accuracy. In contrast, both K-means and Gaussian Mixture assign every spike to a cluster, which often misclassifies ambiguous events. Consequently, HDBSCAN more faithfully represents actual neuronal activity by filtering out only those questionable spikes that impair clarity, surpassing conventional feature-based clustering methods. The spikes recorded with the electrodes shown in the figure can be found at [46], and also at [52], along with the code to analyze them using the different clustering methods. (PDF) [file pbio.3003527.s001.pdf]

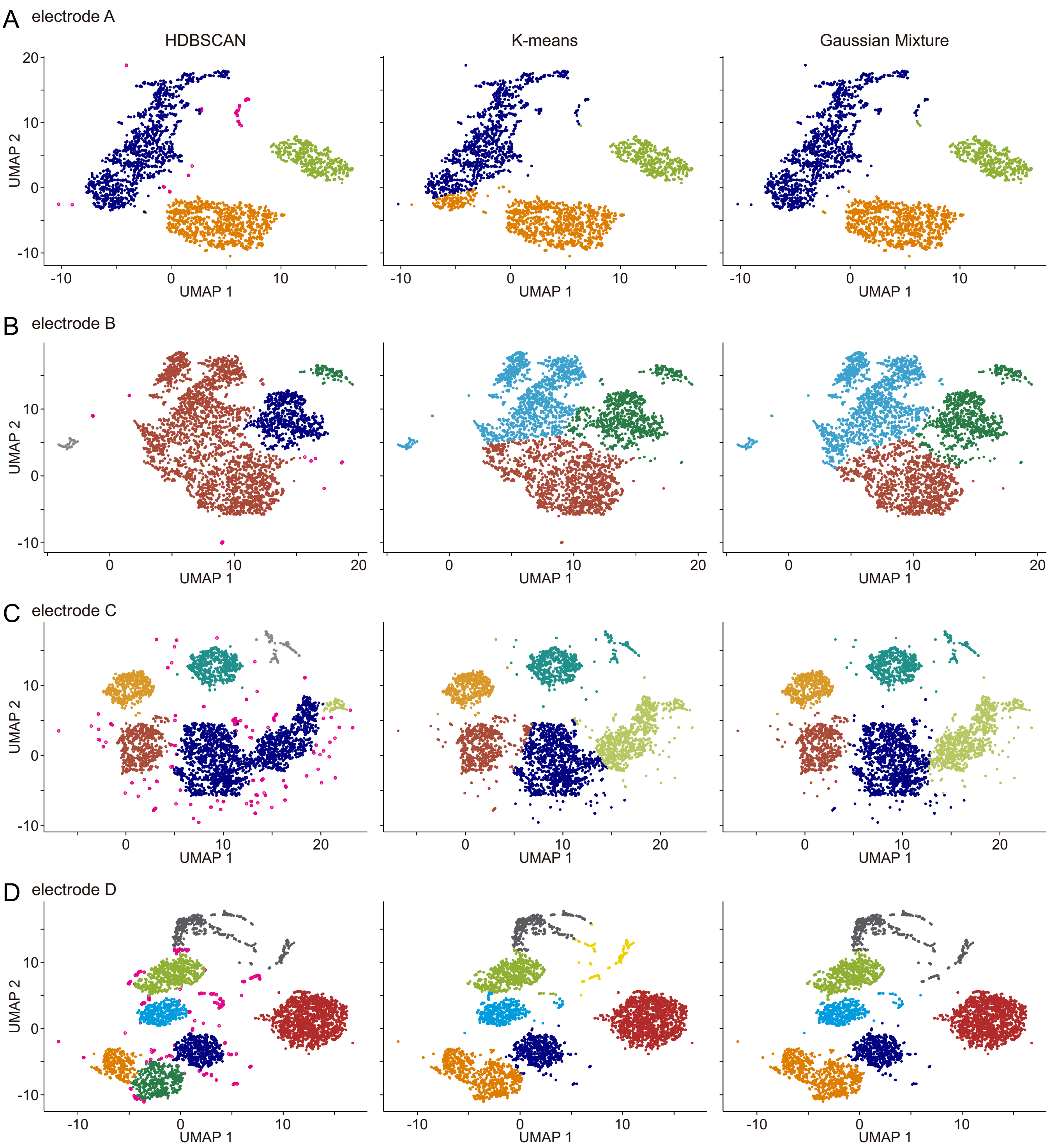

S1 Fig. Comparison of three clustering methods—HDBSCAN, K-means, and Gaussian Mixture—for neuronal spike classification. This panel shows how each approach performs on four electrodes (A–D) during a time interval comparison task, where different colors denote the spikes assigned to putative neurons. HDBSCAN stands out for two key reasons: (1) it can reveal clusters with unusual shapes and densities (see electrodes A–D), and (2) it avoids forcing uncertain spikes (shown in pink) into any cluster. These unassigned points typically represent noise or non-neuronal signals and excluding them sharpens the overall accuracy. In contrast, both K-means and Gaussian Mixture assign every spike to a cluster, which often misclassifies ambiguous events. Consequently, HDBSCAN more faithfully represents actual neuronal activity by filtering out only those questionable spikes that impair clarity, surpassing conventional feature-based clustering methods. The spikes recorded with the electrodes shown in the figure can be found at (46), and also at (52), along with the code to analyze them using the different clustering methods.
