# Supplementary material for: Efficient and reliable spike sorting from neural recordings with UMAP-based unsupervised nonlinear dimensionality reduction
Source: PLoS Biol. 2025 Nov 24;23(11):e3003527. doi: 10.1371/journal.pbio.3003527 (PMC12671831; doi:10.1371/journal.pbio.3003527)
Supplement: S2 Fig — This figure illustrates how different sorting outcomes affect standard performance metrics (precision, recall, and F1 score) when comparing a sorted cluster (Si) against a ground truth (GT) neuron SGT. It also describes the Inclusion Index Matrix (Mi,j). (A–D) Venn diagrams illustrating different scenarios, along with the resulting 2 × 2 Inclusion Matrix visualization when one unit is GT (labeled Ei in the matrix). Precision (Pi = Ii, GT) corresponds to element (1, 2), and recall (Ri = IGT, i) corresponds to element (1, 2). (A) Unrelated/ Worst Case: Low precision and low recall (F1 ≈ 0). Few spikes are shared. (B) High Spike Loss: High precision and low recall. The cluster contains mostly GT spikes but misses many of them (False Negatives). (C) High Contamination: Low precision and high recall. Most GT spikes are captured, but the cluster includes many non-GT spikes (False Positives). (D) Optimal Sorting: High precision and high recall (F1 ≈ 1). The sorted cluster accurately represents the GT neuron. (E) Color Bar for the Inclusion Index values and structure of the Inclusion Index Matrix (Mi,j) for comparing two arbitrary spike trains Si and Sj. (PDF) [file pbio.3003527.s002.pdf]

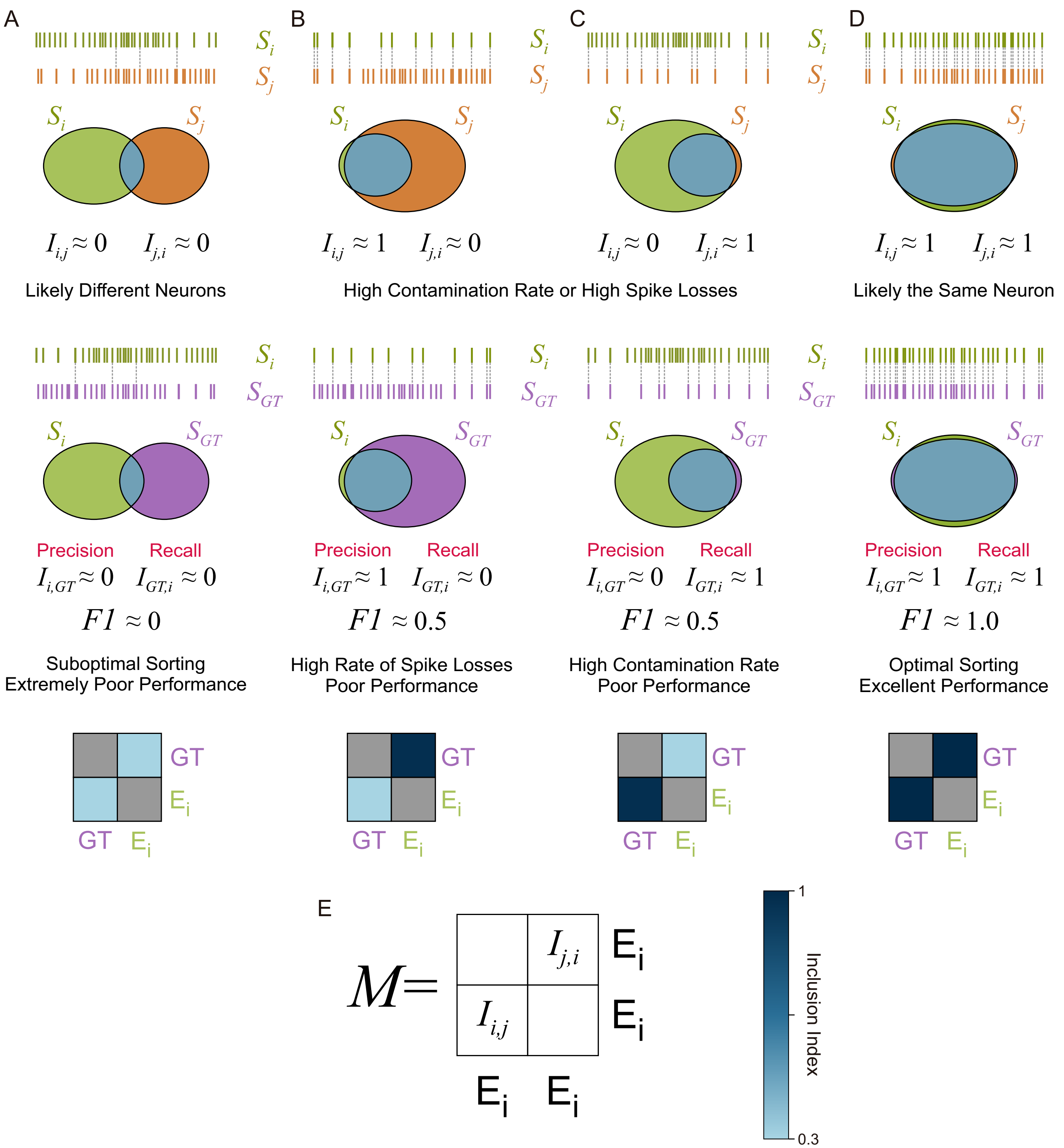

S2 Fig. Schema of spike sorting performance metrics. This figure illustrates how different sorting outcomes affect standard performance metrics (Precision, Recall and F1 score) when comparing a sorted cluster (S_i) against a ground truth (GT) neuron S_{GT}. It also describes the Inclusion Index Matrix ($M_{i,j}$). (A-D) Venn diagrams illustrating different scenarios, along with the resulting 2x2 Inclusion Matrix visualization when one unit is GT (labeled E_i in the matrix). Precision ($P_i = I_{i,GT}$) corresponds to element (2,1), and Recall ($R_i = I_{GT,i}$) corresponds to element (1,2). (A) Unrelated/Worst Case: Low Precision and low Recall (F1 ≈ 0). Few spikes are shared. (B) High Spike Loss: High Precision and low Recall. The cluster contains mostly GT spikes but misses many of them (False Negatives). (C) High Contamination: Low Precision and high Recall. Most GT spikes are captured, but the cluster includes many non-GT spikes (False Positives). (D) Optimal Sorting: High Precision and high Recall (F1 ≈ 1). The sorted cluster accurately represents the GT neuron. (E) Color Bar for the Inclusion Index values and structure of the Inclusion Index Matrix ($M_{i,j}$) for comparing two arbitrary spike trains S_i and S_j.
