# Supplementary material for: Efficient and reliable spike sorting from neural recordings with UMAP-based unsupervised nonlinear dimensionality reduction
Source: PLoS Biol. 2025 Nov 24;23(11):e3003527. doi: 10.1371/journal.pbio.3003527 (PMC12671831; doi:10.1371/journal.pbio.3003527)
Supplement: S3 Fig — This figure illustrates how PCA-, Wavelet -, and UMAP-based methods respond to challenges frequently encountered in spike sorting: overlapping waveforms, background noise, electrode drifting, neuron bursting, and neurons with very low firing rates. All clustering was performed with HDBSCAN. (A, B) PCA (A) and Wavelet (B) projections for an example dataset from Quiroga and colleagues [16]. Colors indicate ground truth (GT) neuron identities, while each marker denotes a detected spike. Although adding higher dimensions can sometimes improve cluster separability, it also complicates visualization, and neither PCA nor Wavelet consistently isolates the three GT neurons. (C) Sorting performance (F1 score) under drifting (left) and bursting (right) conditions. UMAP maintains robust performance (blue trace), whereas PCA (orange trace) and Wavelet (green trace) degrade substantially when waveforms shift over time (drifting) or when neurons exhibit bursts of spikes. (D) Sample spike waveforms with added noise levels (η = 0.05, 0.1, 0.15, 0.2) to synthetic data from Quian Quiroga and colleagues [16]. UMAP-based sorting is robust to increasing background noise. (E) Sorting performance (F1 score) as a function of projection dimensionality at different noise levels (left/middle/right panels for PCA, Wavelet, and UMAP, respectively). At high noise (η = 0.2), UMAP still yields an F1 score close to 0.6 and generally outperforms both PCA and Wavelet. The synthetic data used to generate this figure are publicly available at [40], and the code for performing the analyses is available at [52]. (PDF) [file pbio.3003527.s003.pdf]

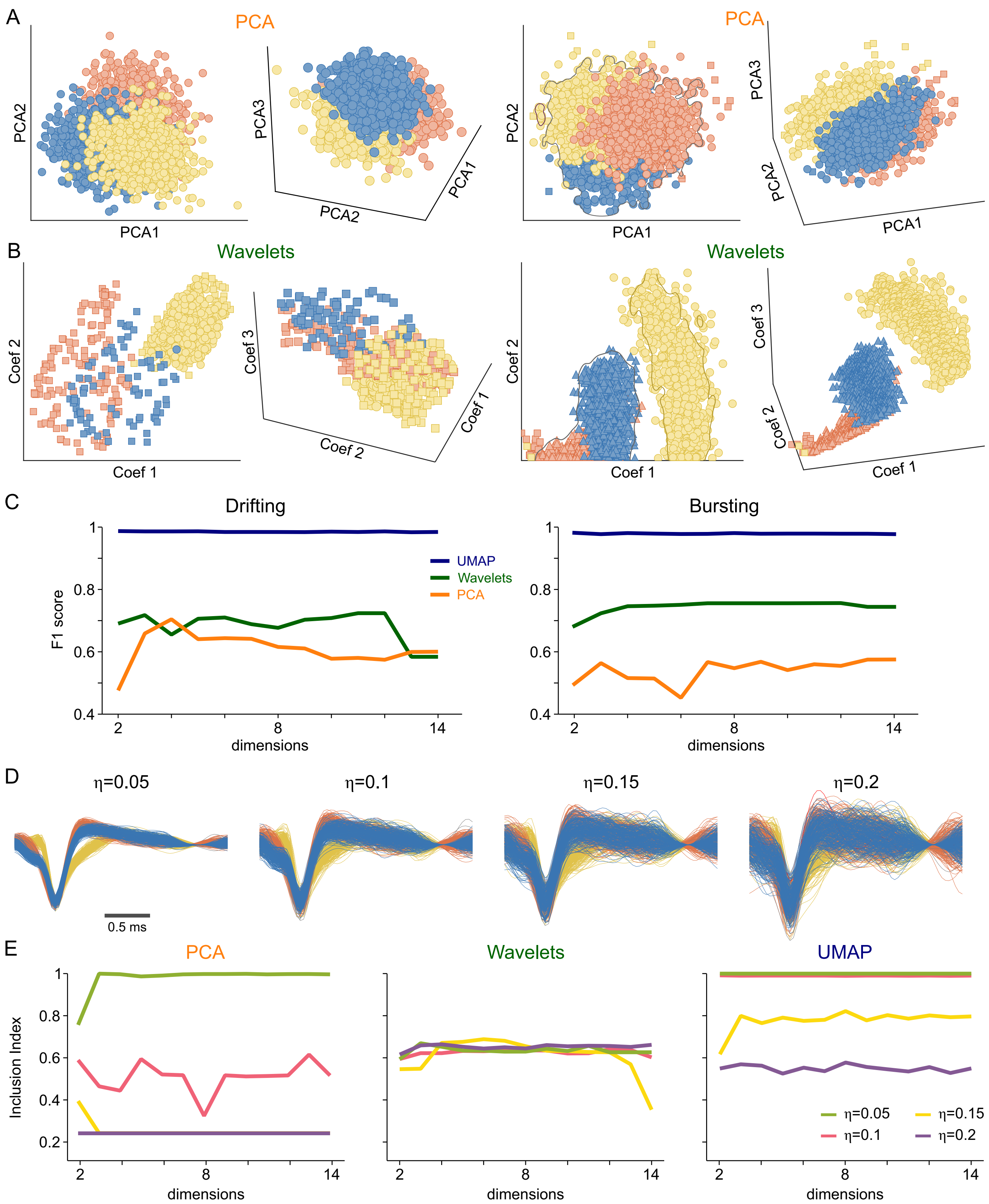
A
PCA
PCA
PCA2
PCA1
PCA3
PCA2
PCA1
PCA2
PCA1
PCA3
PCA2
PCA1
B
Wavelets
Wavelets
Coef 2
Coef 1
Coef 3
Coef 2
Coef 1
Coef 2
Coef 1
Coef 3
Coef 2
Coef 1
C
Drifting
Bursting
F1 score
UMAP
Wavelets
PCA
dimensions
dimensions
D
η=0.05
η=0.1
η=0.15
η=0.2
0.5 ms
E
PCA
Wavelets
UMAP
Inclusion Index
η=0.05
η=0.1
η=0.15
η=0.2
dimensions
dimensions
dimensions

S3 Fig. Impact of noise, drifting, and low firing rates on Spike Sorting performance. This figure illustrates how PCA-, Wavelet -, and UMAP-based methods respond to challenges frequently encountered in spike sorting: overlapping waveforms, background noise, electrode drifting, neuron bursting, and neurons with very low firing rates. All clustering was performed with HDBSCAN. (A, B) PCA (A) and Wavelet (B) projections for an example dataset from Quiroga et al. (16). Colors indicate ground truth (GT) neuron identities, while each marker denotes a detected spike. Although adding higher dimensions can sometimes improve cluster separability, it also complicates visualization, and neither PCA nor Wavelet consistently isolates the three GT neurons. (C) Sorting performance (F1 score) under drifting (left) and bursting (right) conditions. UMAP maintains robust performance (blue trace), whereas PCA (orange trace) and Wavelet (green trace) degrade substantially when waveforms shift over time (drifting) or when neurons exhibit bursts of spikes. (D) Sample spike waveforms with added noise levels (η=0.05, 0.1, 0.15, 0.2) to synthetic data from Quian Quiroga et al. (16). UMAP-based sorting is robust to increasing background noise. (E) Sorting performance (F1 score) as a function of projection dimensionality at different noise levels (left/middle/right panels for PCA, Wavelet, and UMAP, respectively). At high noise (η=0.2), UMAP still yields an F1 score close to 0.6 and generally outperforms both PCA and Wavelet. The synthetic data used to generate this figure are publicly available at (40), and the code for performing the analyses is available at (52).
