# Supplementary material for: Efficient and reliable spike sorting from neural recordings with UMAP-based unsupervised nonlinear dimensionality reduction
Source: PLoS Biol. 2025 Nov 24;23(11):e3003527. doi: 10.1371/journal.pbio.3003527 (PMC12671831; doi:10.1371/journal.pbio.3003527)
Supplement: S4 Fig — The figure shows how pooling spikes from several neurons into a single multiunit can eliminate important task-related encoding—particularly if there is a low-firing-rate neuron. (A) Cartoon showing how poor spike sorting artificially inflates firing rates by combining activity from different neurons, potentially obscuring their distinct encoding patterns. (B) Time Interval Comparison Task (TICT). Animals are comparing two stimulus intervals (Int1 and Int2), both 400–2000 ms, with 2-second gap. The animal must retain Int1 information through the gap and compare it with Int2. Recordings from dorsal premotor cortex (DPC). (C) Raster (top) and firing rate (middle) of a multiunit artificially created by pooling two neurons when using PCA-based sorting. Gray shows the stimulus period; black ticks indicate spikes during correct trials; red ticks indicate spikes during error trials. The mutual information trace (bottom) shows reduced encoding of Int1 since combined units lose valuable signals from low-firing units. (D, E) Single neurons sorted using UMAP that were conflated in (C). While the neuron in (D) fires rarely, however, it does manage to encode Int1 at the start of the delay phase—information lost in the multiunit representation (C). Conversely, the second neuron (E) has a diverging firing pattern and encodes unique dynamics of the task. These two neurons collectively convey far more information about Int1 than the conflated multiunit in (C). The multi-unit and UMAP sorted neuronal activity used to generate the raster plots and firing rates is publicly available at [46], and the code to compute firing rates and mutual information is available at [52]. (PDF) [file pbio.3003527.s004.pdf]

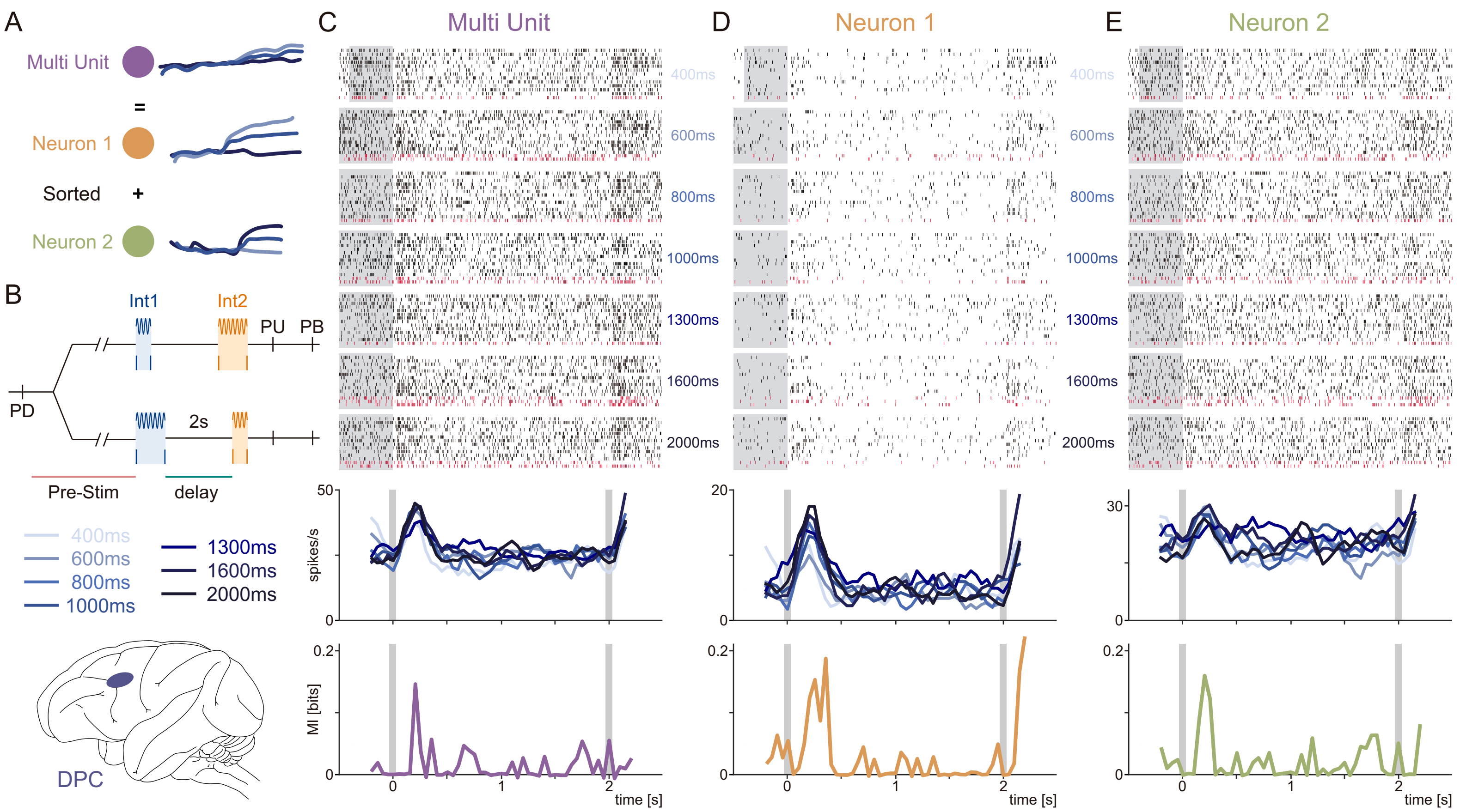

S4 Fig. Preserving low-firing-rate neurons with UMAP sorting in the Time Interval Comparison Task. The figure shows how pooling spikes from several neurons into a single muti-unit can eliminate important task-related encoding—particularly if there is a low firing rate neuron. (A) Cartoon showing how poor spike sorting artificially inflates firing rates by combining activity from different neurons, potentially obscuring their distinct encoding patterns. (B) Time Interval Comparison Task (TICT). Animals are comparing two stimulus intervals (Int1 and Int2), both 400–2000 ms, with 2-second gap. The animal must retain Int1 information through the gap and compare it with Int2. Recordings from dorsal premotor cortex (DPC). (C) Raster (top) and firing rate (middle) of a multi-unit artificially created by pooling two neurons when using PCA-based sorting. Gray shows the stimulus period; black ticks indicate spikes during correct trials; red ticks indicate spikes during error trials. The mutual information trace (bottom) shows reduced encoding of Int1 since combined units lose valuable signals from low-firing units. (D, E) Single neurons sorted using UMAP that were conflated in (C). While the neuron in (D) fires rarely, however, it does manage to encode Int1 at the start of the delay phase—information lost in the multi-unit representation (C). Conversely, the second neuron (E) has a diverging firing pattern and encodes unique dynamics of the task. These two neurons collectively convey far more information about Int1 than the conflated multi-unit in (C). The multi-unit and UMAP sorted neuronal activity used to generate the raster plots and firing rates is publicly available at (46), and the code to compute firing rates and mutual information is available at (52).
