# Supplementary material for: Efficient and reliable spike sorting from neural recordings with UMAP-based unsupervised nonlinear dimensionality reduction
Source: PLoS Biol. 2025 Nov 24;23(11):e3003527. doi: 10.1371/journal.pbio.3003527 (PMC12671831; doi:10.1371/journal.pbio.3003527)
Supplement: S5 Fig — (A) Schematic showing how inadequate spike sorting inflates firing rates by combining separate neuronal activities, potentially masking each neuron’s individual encoding. (B) Tactile detection task: Animals receive a vibrotactile stimulus (0–24 μm) on the fingertip and must indicate whether it was present or absent. Recordings are from the ventral premotor cortex (VPC, green). (C) Raster plots (top) and firing rate (bottom) for a multiunit formed by merging two neurons under PCA-based sorting. Trials are aligned to stimulus onset. Blue shading marks stimulus-present trials; gray shading marks stimulus-absent trials. Combining these spikes conceals each neuron’s separate response profile, yielding an incomplete picture of their activities. (D, E) UMAP-sorted versions of the two underlying neurons from (C). The neuron in (E) maintains a prolonged response during the delay, while the neuron in (D) responds strongly but briefly. By separating them, UMAP-based sorting retains each neuron’s individual activity, avoiding the loss of valuable low-firing-rate signals. The multiunit and UMAP sorted neuronal activity used to generate the raster plots and firing rates is publicly available at [46], and the code to compute firing rates and mutual information is available at [52]. (PDF) [file pbio.3003527.s005.pdf]

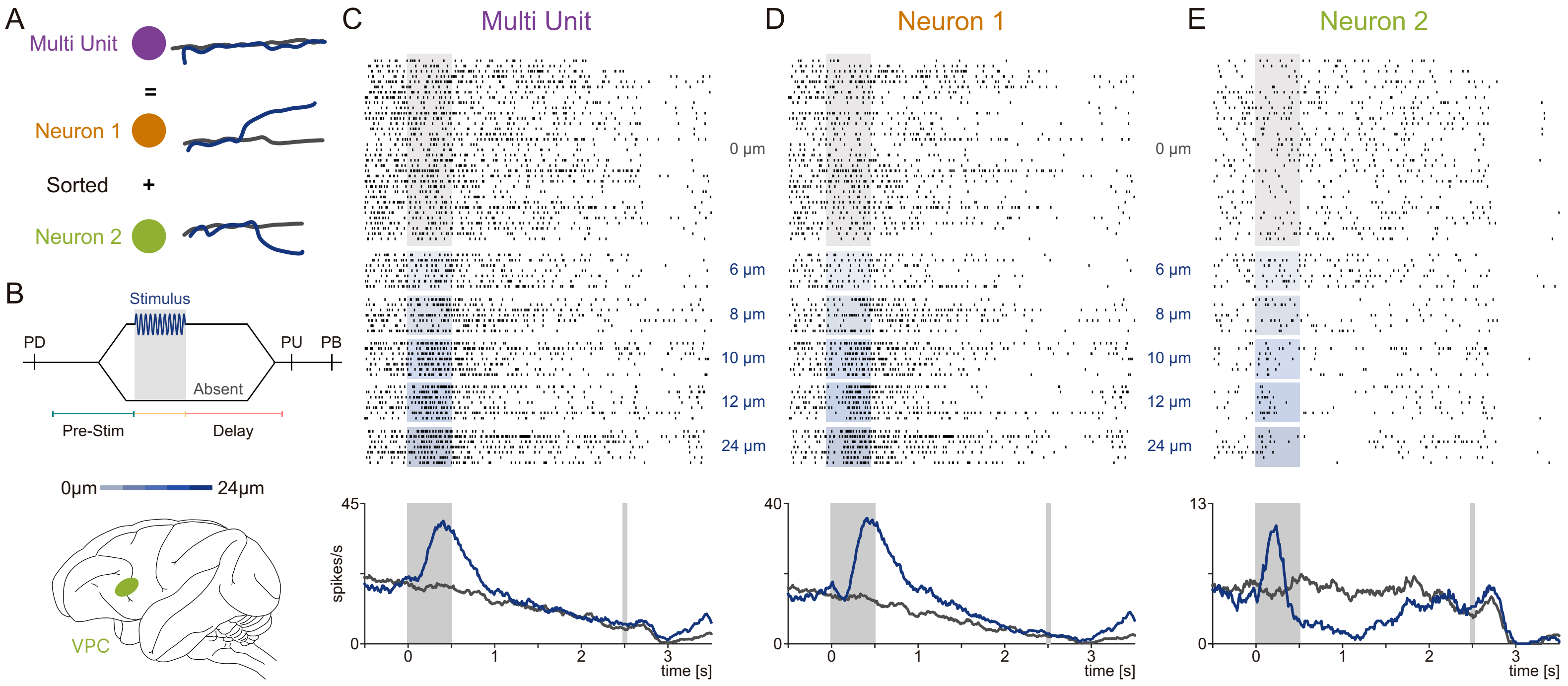

S5 Fig. Preserving different encoding patterns with UMAP-based sorting in tactile detection task. (A) Schematic showing how inadequate spike sorting inflates firing rates by combining separate neuronal activities, potentially masking each neuron's individual encoding. (B) Tactile detection task: Animals receive a vibrotactile stimulus (0–24 µm) on the fingertip and must indicate whether it was present or absent. Recordings are from the ventral premotor cortex (VPC, green). (C) Raster plots (top) and firing rate (bottom) for multi-unit formed by merging two neurons under PCA-based sorting. Trials are aligned to stimulus onset. Blue shading marks stimulus-present trials; gray shading marks stimulus-absent trials. Combining these spikes conceals each neuron's separate response profile, yielding an incomplete picture of their activities. (D, E) UMAP-sorted versions of the two underlying neurons from (C). The neuron in (E) maintains a prolonged response during the delay, while the neuron in (D) responds strongly but briefly. By separating them, UMAP-based sorting retains each neuron's individual activity, avoiding the loss of valuable low-firing-rate signals. The multi-unit and UMAP sorted neuronal activity used to generate the raster plots and firing rates is publicly available at (46), and the code to compute firing rates and mutual information is available at (52).
