# Supplementary material for: Efficient and reliable spike sorting from neural recordings with UMAP-based unsupervised nonlinear dimensionality reduction
Source: PLoS Biol. 2025 Nov 24;23(11):e3003527. doi: 10.1371/journal.pbio.3003527 (PMC12671831; doi:10.1371/journal.pbio.3003527)
Supplement: S6 Fig — (A) Sorting performance (F1 score) versus distance for three example MEAs using single-channel UMAP sorting. (B) Spatial map of the F1 score across the MEA for the corresponding examples in A. Black squares indicate the region shown in C. (C) Waveform templates from the neuron with the highest F1 score, shown across the 5x5 electrode patch highlighted in (B). The largest amplitude waveform is recorded on the central electrode (closest to the GT), and the color saturation of each waveform corresponds to the local F1 score. UMAP robustly identifies the characteristic waveform across multiple neighboring electrodes. MEA recordings supporting the analyses are available at [42], and the code necessary for the analyses is available at [52]. (PDF) [file pbio.3003527.s006.pdf]

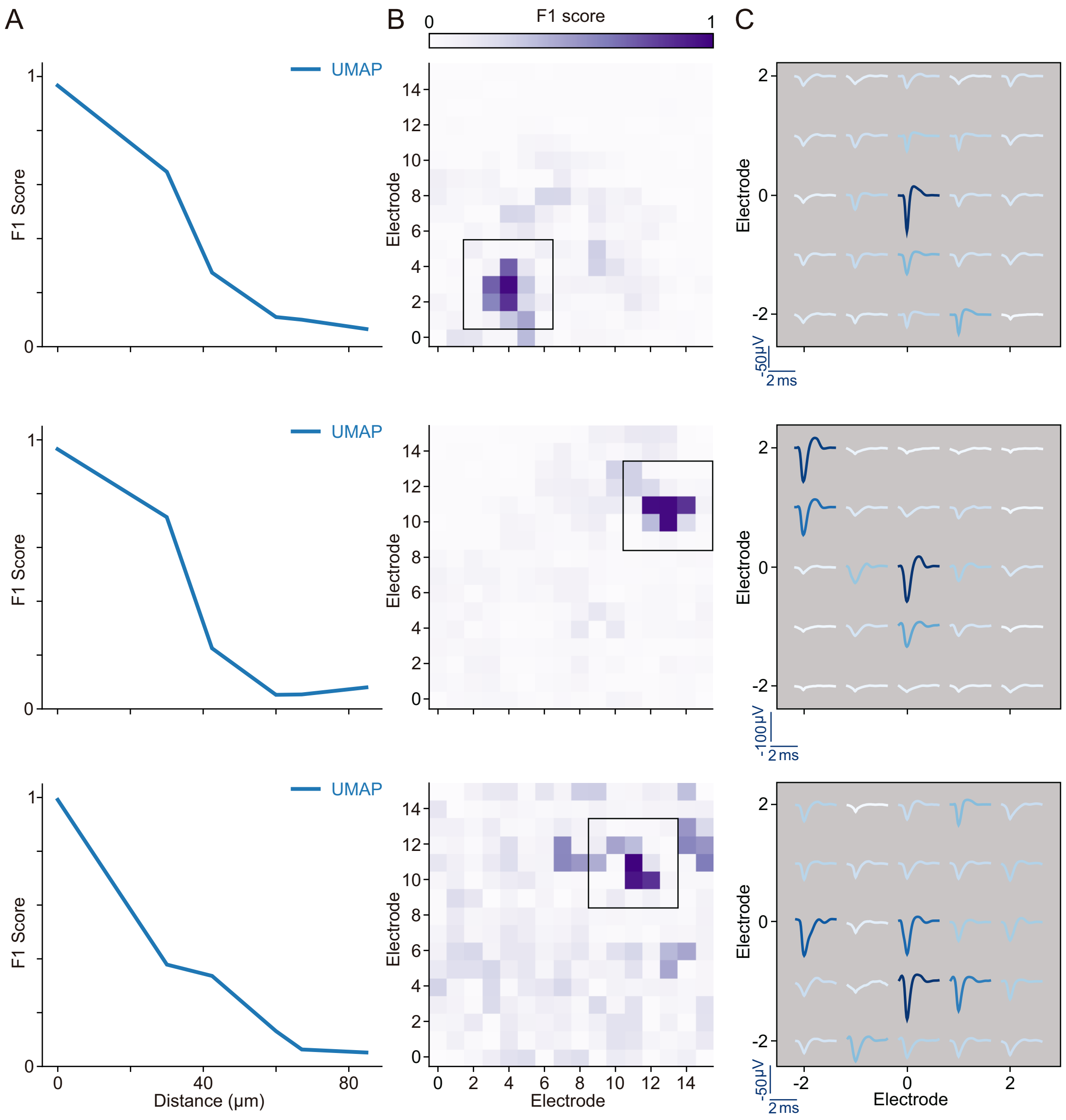

S6 Fig. Examples of UMAP spatial robustness in MEA recordings. (A) Sorting performance (F1 score) vs. distance for three example MEAs using single-channel UMAP sorting. (B) Spatial map of the F1 score across the MEA for the corresponding examples in A. Black squares indicate the region shown in C. (C) Waveform templates from the neuron with the highest F1 score, shown across the 5x5 electrode patch highlighted in (B). The largest amplitude waveform is recorded on the central electrode (closest to the GT), and the color saturation of each waveform corresponds to the local F1 score. UMAP robustly identifies the characteristic waveform across multiple neighboring electrodes. MEA recordings supporting the analyses are available at (42), and the code necessary for the analyses is available at (52).
