# Supplementary material for: Efficient and reliable spike sorting from neural recordings with UMAP-based unsupervised nonlinear dimensionality reduction
Source: PLoS Biol. 2025 Nov 24;23(11):e3003527. doi: 10.1371/journal.pbio.3003527 (PMC12671831; doi:10.1371/journal.pbio.3003527)
Supplement: S7 Fig — (A–F) Six examples showing the decay of F1 score as a function of distance from the central electrode. The solid blue line (UMAP) tracks performance as signals from neighboring electrodes at progressively greater distances (d1 → d6) are incorporated into the analysis. For comparison, dashed lines show the overall F1 score value when using the entire multielectrode array (MEA) signal at once for both UMAP (dashed blue) and SpyKING CIRCUS (SC, dashed light blue). UMAP consistently outperforms SC, particularly when leveraging this spatial information. MEA recordings supporting the analyses are available at [42], and the code necessary for the analyses is available at [52]. (PDF) [file pbio.3003527.s007.pdf]

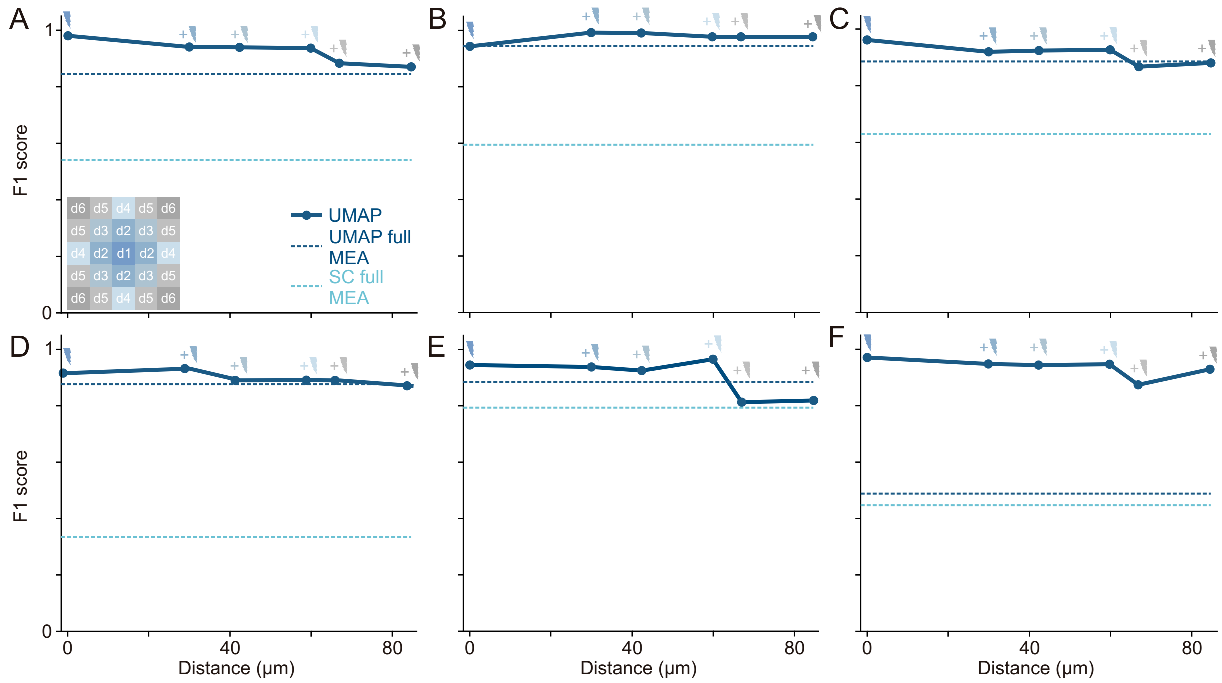

S7 Fig. Geometric analysis examples for multi-electrode UMAP sorting. (A-F) Six examples showing the decay of F1 score as a function of distance from the central electrode. The solid blue line (UMAP) tracks performance as signals from neighboring electrodes at progressively greater distances (d1→d6) are incorporated into the analysis. For comparison, dashed lines show the overall F1 score value when using the entire MEA signal at once for both UMAP (dashed blue) and SpyKING CIRCUS (dashed light blue). UMAP consistently outperforms SC, particularly when leveraging this spatial information. MEA recordings supporting the analyses are available at (42), and the code necessary for the analyses is available at (52).
