# Supplementary material for: Efficient and reliable spike sorting from neural recordings with UMAP-based unsupervised nonlinear dimensionality reduction
Source: PLoS Biol. 2025 Nov 24;23(11):e3003527. doi: 10.1371/journal.pbio.3003527 (PMC12671831; doi:10.1371/journal.pbio.3003527)
Supplement: S8 Fig — This figure examines how precision (related to contamination) and recall (related to lost spikes) contribute to the spatial decay of the F1 score (Fig 5) when comparing Uniform Manifold Approximation and Projection (UMAP) and SpyKING CIRCUS (SC) on MEA recordings. Results are averaged over multiple MEAs. (A, D) Average spatial matrices for precision (A) and recall (D) computed using UMAP-based sorting. (B, C) Decay of precision, and fit of that decay (C), as electrode distance increases for UMAP. Triangles on (B) represent average precision values computed with UMAP-based sorting (light-blue) or SC (cyan) using the entire MEA signal. (E, F) Decay of recall, and fit of that decay (F), as electrode distance increases. Triangles on (E) represent average recall values computed with UMAP-based sorting (orange) or SC (yellow) using the entire MEA signal. Note that average for recall using SC is significantly low with respect to UMAP’s, indicating the loss of many GT spikes beyond the central electrode. By contrast, UMAP sustains higher precision and recall across a wider spatial range. MEA recordings supporting the analyses are available at [42], and the code necessary for the analyses is available at [52]. (PDF) [file pbio.3003527.s008.pdf]

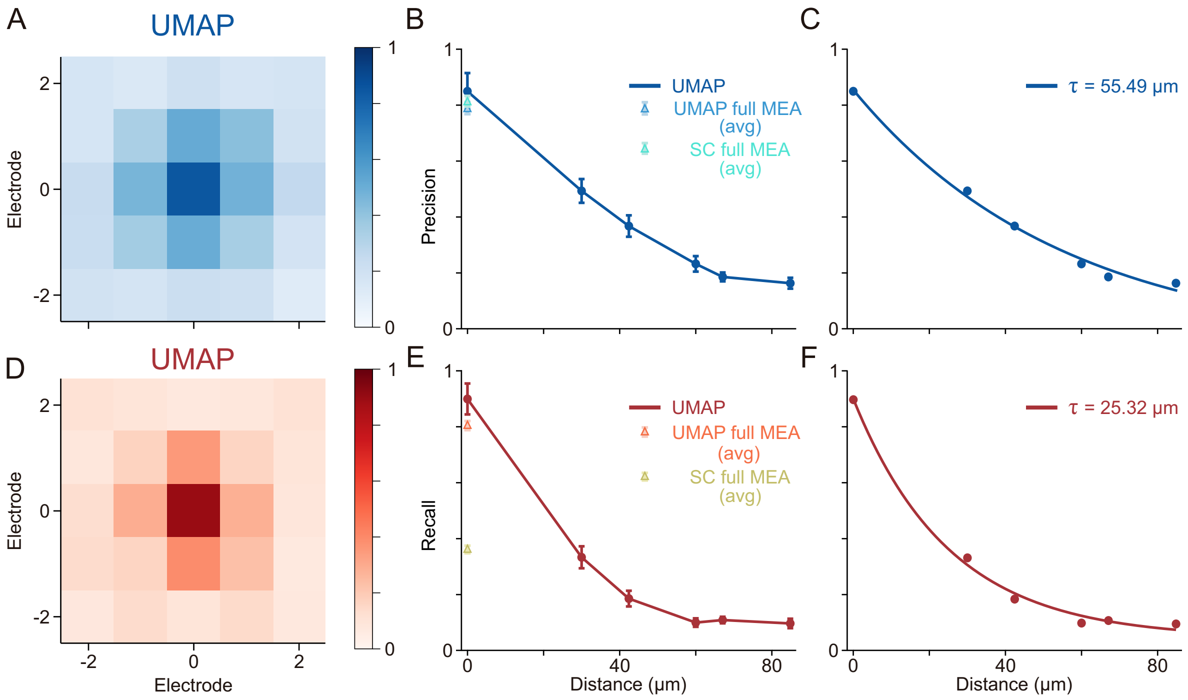

S8 Fig. Influence of Precision and Recall on MEA sorting accuracy. This figure examines how Precision (related to contamination) and Recall (related to lost spikes) contribute to the spatial decay of the F1 score (Fig. 5) when comparing UMAP and SpyKING CIRCUS (SC) on MEA recordings. Results are averaged over multiple MEAs. (A, D) Average spatial matrices for Precision (A) and Recall (D) computed using UMAP-based sorting. (B, C) Decay of Precision, and fit of that decay (C), as electrode distance increases for UMAP. Triangles on (B) represent average Precision values computed with UMAP-based sorting (light-blue) or SC (cyan) using the entire MEA signal. (E, F) Decay of Recall, and fit of that decay (F), as electrode distance increases. Triangles on (E) represent average Recall values computed with UMAP-based sorting (orange) or SC (yellow) using the entire MEA signal. Note that average for Recall using SC is significantly low with respect to UMAP's indicating the loss of many GT spikes beyond the central electrode. By contrast, UMAP sustains higher Precision and Recall across a wider spatial range. MEA recordings supporting the analyses are available at (42), and the code necessary for the analyses is available at (52).
