# Supplementary material for: Efficient and reliable spike sorting from neural recordings with UMAP-based unsupervised nonlinear dimensionality reduction
Source: PLoS Biol. 2025 Nov 24;23(11):e3003527. doi: 10.1371/journal.pbio.3003527 (PMC12671831; doi:10.1371/journal.pbio.3003527)
Supplement: Attachment — Submitted filename: Reply to reviewers - Suarez-Barreda et al.pdf [file pbio.3003527.s011.pdf]

We sincerely thank the editor and reviewers for their valuable feedback and insightful comments, which have significantly contributed to the improvement of our manuscript's quality. Below, we provide detailed responses to each of the reviewers' suggestions.

Responses to Reviewer #1

Reviewer #1 (Alessio Paolo Buccino): *The authors of this manuscript developed a UMAP-based spike sorting pipeline and benchmarked its performance against other methods on simulated and experimental ground-truth data.*
The manuscript also shows that UMAP outperforms other dimensionality-reduction methods, especially in its ability to find low-firing units.
While the methodology is well described and sound, I think that there are some major points that need further work and clarification.

We sincerely thank the reviewer for their insightful and constructive feedback, which has significantly improved the rigor and clarity of our manuscript. We have addressed all the major and minor concerns raised. In particular, we have conducted substantial new analyses to address the critique regarding multi-electrode recordings and have revised the manuscript to adopt standard performance metrics throughout.

Major issues

1. Multi-electrode recordings:
At several places, the authors claim that the method can improve spike sorting performance of high-density multi-electrode arrays. However, they present no results where the developed pipeline (and other methods) use the additional spatial information provided by multi-electrode probes.
In the last two sections of the results ("Comparing UMAP-based sorting and SpyKING CIRCUS in multielectrode array recordings: the role of distance" and "Comparing UMAP-based and Feature-based spike sorting in multi-electrode recordings"), the authors use multi-electrode data, but analyze the recording of each electrode separately.
This defeats the purpose of multi-electrode data, where the spatial redundancy is exactly what is used by different methods to refine the spike sorting. Spyking CIRCUS, for example, uses data from multiple electrodes both for extracting features and for template matching. The analysis that they provide and the comparison with Spyking CIRCUS is therefore limited and unfair in my opinion, and should be removed. Otherwise, the authors should let Spyking CIRCUS use all the available electrodes, since this is its intended use.

Similarly, the data from Buszaki is from tetrodes that were developed primarily to increase the separability of neurons by recording them on multiple closed-by electrodes. For these reasons, I think that the authors should either add a multi-electrode implementation, e.g., where features from different electrodes are combined prior to clustering, or they should reframe the "multi-electrode" claims, and state that the method is primarily designed (as it stands) for single electrode/microwire (and could be extended to multi-channel data).
I think that this could still be relevant, and the analysis of distance-related performance (in the comparison with Spyking CIRCUS) could further provide evidence on the fact that UMAP can work well on low SNR regimes, especially in relation to PCA and Wavelet methods.

We fully agree with the reviewer that a fair comparison requires evaluating both algorithms in their intended use cases, utilizing the spatial information inherent in MEA recordings. The reviewer correctly pointed out that our initial analysis, which processed electrodes independently, was limited and did not adequately support our claims regarding high-density arrays. Following the reviewer's suggestion, we have implemented and thoroughly tested multi-electrode versions of the UMAP pipeline and have substantially revised the corresponding sections of the manuscript.

1. Implementation of Multi-electrode UMAP strategies: We developed strategies to allow UMAP to leverage spatiotemporal information by concatenating features from multiple electrodes prior to dimensionality reduction (detailed in the revised Methods section, "*Multi-electrode array (MEA) analysis and spatial accuracy*"). We implemented two specific approaches:

- **Geometric Analysis (Cumulative):** We progressively incorporated features from concentric rings of electrodes centered on the ground truth (GT) neuron hotspot (please see schema on Fig. 5E and S7A, dark blue traces on Figs. 5E, 5F and S7A-F).
- **Full MEA (Simultaneous):** We concatenated features from all available electrodes across the MEA simultaneously. Represented as dark and light-blue triangles for UMAP and SpyKING CIRCUS, respectively, on Figs. 5C (single MEA values), 5D (average across all MEAs). Same values are represented in Figs. 5E as dark and light-blue dashed lines and in 5F with the same color pattern but with long dashed lines representing the average across all MEAs (as triangles in Fig. 5D).

2. Fair comparison with SpyKING CIRCUS (SC): We compared these multi-channel UMAP approaches against SpyKING CIRCUS, which utilized all available electrodes (SC full MEA), as intended. **<u>Importantly:</u>** we deeply apologized with the reviewer if this was

not correctly stated in the original manuscript; we have ALWAYS run SC using the entire signal of the recording.

Analyses using SC represented on panels C, D, E and F in the original Fig. 5 were conducted by running SC over the entire MEA signal and then we ordered the sorted spikes times with respect to their corresponding electrodes, therefore we were able to compute the contribution of each electrode to F1 score, Recall and Precision.

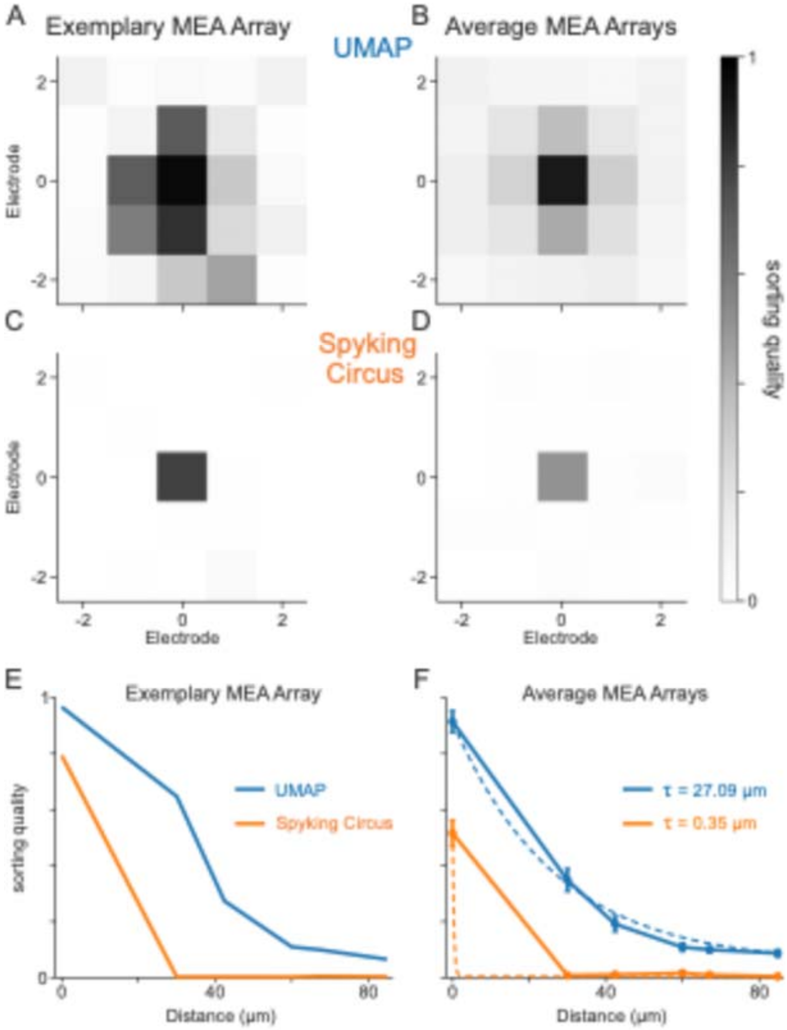

However, given that SC processes the whole signal at once, we agree with the reviewer that it is pointless and unfair to conduct a comparative decay (F1 score vs distance) analysis between SC and the UMAP-based sorting, when the latest is only applied over each single electrode. Accordingly, we have substantially revised **Figure 5**. The decay curves for SC have been removed (orange traces). The comparison between the UMAP pipeline and SC is now performed under equitable conditions: both algorithms are applied to the full MEA signal simultaneously. The results of this direct, full-array comparison are now shown as **triangles** in the revised **Fig. 5C, D** and **dashed lines Fig. 5E, F** (overall **F1 score**). We also represent overall F1 score values with dashed lines for

SC and UMAP-based sorting on **Fig. S7A-F** and in **Figure S8B, E** we represented with triangles the overall values for **Precision** and **Recall**, also employing SC and the UMAP-based sorting. The updated figures now better highlights our primary analysis of how the UMAP-based pipeline effectively leverages spatial information.

3. New findings and revisions (Figure 5): The new results, presented in the revised Figure 5 (and new supplementary figures S6, S7), robustly demonstrate the capability of the UMAP-based approach in leveraging spatial information.

- **Performance:** When utilizing spatial information (either progressively via geometric analysis or simultaneously), UMAP shows an outstandingly performance (Fig. 5A-F and Fig. S7A-F), also outperforming SC when the comparison is being made using the entire MEA signal (triangles on Fig. 5C, D and dashed lines on Fig. S7A-F).

- **Enhanced spatial robustness:** UMAP exhibits a quantitatively slow decay in performance as the distance from the neuron increases ($\tau \approx 27.09$ µm) (Fig. 5D).

- **Robustness in low Signal-Noise-Ratio (SNR):** We retained the single-channel analysis, as suggested by the reviewer, to demonstrate UMAP's intrinsic robustness in low-SNR regimes (Fig. 5 A-D), where it is highlighted the strength of the nonlinear embedding even without explicit spatial information.

We have revised the Results section "*Comparing UMAP-based sorting and SpyKING CIRCUS in multielectrode array recordings: the role of spatial information*" (Pages 12-14) the Discussion (Page 17, paragraph 4) and Methods (Pages 21-22), to reflect these new findings.

4. Clarification of the Buzsaki dataset analysis (Figure 6): Regarding the Buzsaki tetrode data, we acknowledge that this data is also intended for multi-channel analysis. However, following the reviewer's insight that analyzing distance-related performance can provide evidence of robustness in low-SNR regimes, we have retained the independent analysis of these electrodes. We have clarified the rationale in the text ("*Comparing UMAP-based and Feature-based spike sorting in multi-electrode recordings*"), framing this analysis as a complementary test of the intrinsic robustness of the dimensionality reduction methods (UMAP, PCA, Wavelet), independent of spatial integration techniques (Results, Page 14).

We believe these substantial revisions and new analyses directly address the reviewer's concerns and strongly support our claims regarding the suitability and high performance of UMAP-based sorting for multi-electrode array recordings. We are sincerely grateful to the reviewer for their constructive feedback, which has helped us significantly strengthen our manuscript and its findings.

2. Performance metrics.

The authors define additional metrics and names for measuring performance. Only in the methods, they explain that Igt,i is equivalent to recall and Ii,gt is equivalent to precision. They further define an overall quality as the average between precision and recall.

I think that this new terminology is not needed and it makes the reading more confusing. Instead, I suggest to use existing metrics such as precision, recall throughout the manuscript. The quality score is not robust against imbalanced between precision and recall.

I believe that using more standard metrics, such as accuracy and F1 score would be more familiar to readers and overall improve the clarity of the manuscript.

We thank the reviewer for this important suggestion. We agree that using standard, well-established metrics improves clarity and robustness.

We have revised the entire manuscript to replace our previous terminology ($I_{i,GT}$, $I_{GT,i}$, and Sorting Quality $Q_{i,GT}$) with the standard metrics: **Precision**, **Recall**, and **F1 score** (the harmonic mean of Precision and Recall), respectively.

<u>**Specific changes include:**</u>

1. **Results section:** The section "*Spike sorting performance metrics*" (Page 7) has been rewritten to define and utilize F1 score, Precision, and Recall. These metrics are now used consistently in all subsequent Results sections.
2. **Methods section:** The section "*Performance Metrics and Inclusion Index Matrix*" (Page 21) has been updated to formally define F1 score, Precision, and Recall based on True Positives, False Positives, and False Negatives.
3. **Figures and Legends:** We have updated all relevant figures and their legends to use the standard terminology. This includes Figures 2, 5, 6, and Supplementary Figures S2, S3, S6, S7, and S8. Figure S2 has been revised to clearly illustrate how different sorting scenarios map onto Precision, Recall, and F1 score.

We believe these changes significantly improve the clarity of the manuscript and align our evaluation methodology with community standards.

Minor issues

1. Title: Nonlinear Dimensionality Reduction can be any method. I suggest changing it to UMAP.

We thank the reviewer for their insightful suggestion. Now manuscript's new title is:

*“**Relevance of UMAP for efficient and robust spike sorting**”*

2. Introduction: silicon -> silicon probes

3. Introduction: Herding -> Herding Spikes

4. Introduction: different neurons) -> different neurons,

5. Introduction: These reasons is why -> We have restructured the corresponding paragraph, and the sentence in question has been removed.

We thank the reviewer for their constructive and helpful suggestions, and the opportunity to improve the quality of our manuscript. All typos have been corrected.

8. Introduction: "As the number of electrodes in an array increases, methods that require parameter hand-tuning for guaranteed performance become increasingly less usable." What methods require hand tuning? All the presented methods (PCA/Wavelet) are fully automatic.

Thank you for this excellent point, which allows us to highlight a key advantage of our pipeline. We agree that many sorting pipelines are not fully automatic because they rely on clustering algorithms that require manual parameter tuning—most notably, pre-specifying the number of clusters. To specifically overcome this limitation, we selected HDBSCAN for the clustering step. A central advantage of HDBSCAN is that it automatically determines the optimal number of clusters from the data's density structure, making such manual tuning unnecessary. This choice is crucial for making our pipeline more robust and end-to-end automatable, and we have revised the manuscript to emphasize this advantage more clearly.

<u>To improve clarity the current sentence has now been modified to</u>:

> *“As the number of electrodes in an array increases, methods that require parameter hand-tuning (specifying the number of sought spike clusters) or extensive manual curation for guaranteed performance become increasingly unsuitable.”*

9. Introduction: "…using a 64-electrode multiarray throughout a week of recordings generates more than 300 different datasets, each consisting of a variable number of identifiable neurons". Why is that? How did the authors come up with 300? One could record continuously for a full week…

We appreciate the opportunity to clarify this point. Our estimate is for acute recordings where data from each of the 64 channels are treated as distinct datasets. Following this, an experimental week with five recording sessions would produce approximately 320 datasets (64 channels/session × 5 sessions). The manuscript has been updated to better explain this assumption.

To improve clarity the current sentence has now been modified to:

> *"For example, assuming multiple acute sessions per day across a week—resulting in, say, 5 sessions per electrode per week—the use of a 64-electrode multiarray generates approximately 320 (i.e., 64 electrodes recordings times 5 sessions) independent extracellular activity datasets, each containing a variable and unknown number of putative neurons, as a function of largely uncontrolled session-specific conditions."*

10. Introduction: What do the authors mean with "expert-defined" metrics?

Thanks for the question, in this context we are referring to particular metrics as width of the waveform, peak to peak amplitude, etc. We have extended that paragraph to be explicit; the current paragraph shows as follows:

> *"... Conversely, spike sorting pipelines based on linear dimensionality reduction methods or expert-defined metrics, like peak-to-peak amplitude, temporal width, or axis length in the phase space, just to name a few… "*

11. Results: "UMAP-based spike sorting vs. traditional feature-based pipelines" UMAP is also feature-based. I would drop "feature-based" and just leave "traditional pipelines"

We thank the reviewer for their suggestion and the opportunity to improve the precision and coherence of our manuscript. We have changed the title of the mentioned section, which now it should be read as:

> ***"UMAP-based spike sorting vs. traditional pipelines"***

12. Results: (ranging from 0.05 to 0.2) what are the units of this?

We appreciate the reviewer's comment and the opportunity to improve our manuscript. We apologize for the lack of clarity in this point. In this scenario η represents the standard deviation of the background noise relative to the amplitude of the spike waveforms.

We have now included this definition in the third paragraph of the subsection "*UMAP-based spike sorting achieves superior and robust performance*" in the Results:

> "*Because noise is a critical factor in neural spike sorting, we explored how UMAP handles increasing noise levels (η ranging from 0.05 to 0.2, where η represents the standard deviation of the background noise relative to the amplitude of the spike waveforms (16)) added to three synthetic spike waveforms from (16) (Fig. S3D), and compare it against PCA and Wavelet decomposition (Fig. S3E, right, middle, and left panels, respectively).*"

We have also modified this definition in Methods, in the second paragraph of the subsection "*Comparative analysis of different databases*":

> "*... eight noise levels ranging from 0.05 to 0.4 with increments of 0.05, while the other three datasets included four noise levels ranging from 0.05 to 0.2. As described in the original work, noise level is represented in terms of its standard deviation relative to the peak amplitude of the spikes. All spike classes had a peak value of 1.*"

Furthermore, we have added a new subsections methods named "*Synthetic data simulations*", where we have included these definitions:

> "***Noise and low firing rates:*** *Background noise (η) is defined as the standard deviation of the background noise relative to the amplitude of the spike waveforms (16). Following the methodology of (16), this noise is modeled as structured (colored) noise, generated by summing spike waveforms from non-sorted background unit at random times. To simulate low firing rates (silent neurons), we randomly subsampled one of the ground truth clusters.*"

13. Results: " we randomly removed an increasing fraction of the datapoints from one of the synthetic GT neuron clusters". What is the original firing rate? How are spikes removed and what are the "final" firing rates?

We thank the reviewer for this suggestion, which has strengthened our manuscript. Accordingly, we have revised the third paragraph of the subsection "*UMAP-based spike sorting achieves superior and robust performance*" in the Results. The text now explicitly states that spikes were randomly removed and includes the original parameters of the simulation, such as the synthetic neurons' constant 20 Hz firing rate and the initial cluster size:

> *"Finally, to test the ability of UMAP-based sorting to detect "silent" neurons, we randomly removed an increasing fraction of the data points from one of the synthetic GT neuron clusters (the red one, as per Fig. 2A). Crucial, in this synthetic dataset all the neurons have a 20 Hz constant rate, then after random spike removal, spiking activity is not constant anymore. Remarkably, UMAP still ensured almost perfect (~1) F1 score when only 40% of the cluster data points were retained and still very good (~0.8) F1 score even when only 20% of the data points were retained."*

14. Results: "thereby increasing the average number of neuron pairs from about 28 to almost 70." Why is this relevant? I think it's enough to report the increased number of identified neurons.

We appreciate the opportunity to clarify this point. The increase in neuron pairs is highlighted because it directly impacts the feasibility and power of analyses focused on pairwise interactions, which are critical for understanding population codes. The key is that the number of neuron pairs does not scale linearly but quadratically with the neuron count. Therefore, the modest increase in simultaneously recorded neurons (from roughly 8 to 13) yields a much more significant, ~2.5-fold increase in the number of pairs available for correlation analysis (from 28 to almost 70). We believe this point is relevant as it underscores how our method provides richer datasets for studying neural interactions.

15. Results: is ablet -> is able

We apologize for the typographical errors and thank the reviewer for their careful reading. The manuscript has been corrected accordingly.

16. Discussion: Reference 47 (SimSort) showed a deep-learning based method that outperforms UMAP. You should comment on that.

We thank the reviewer for this valuable suggestion. As recommended, we have revised the final paragraph of the Discussion to contextualize our method with recent deep-learning approaches like SimSort. The new text highlights the advantages of our unsupervised pipeline as follows:

> *"Relative to other contemporary spike sorting approaches with UMAP, like WaveMAP (28) (used for cell type classification), which involves graph-based clustering, or P-sort (41) (a spike sorting method tailored for the cerebellum), our method utilizes only UMAP along with HDBSCAN clustering. While recent deep-learning methods like SimSort (16, 17, 49) have demonstrated excellent performance by leveraging extensive training on simulated data, our approach provides a powerful, unsupervised alternative that does not require prior training*

or assumptions about waveform shapes. This direct and efficient method obviates additional preprocessing or specialized clustering algorithms, thus enhancing computational efficiency, enabling automation, and increasing generalizability to various types of neurons and experimental conditions."

17. Discussion / Methods: "Drifting" usually refers to the movement between electrodes/neurons, which causes a variation in spike shape over time. I would use something less misleading, like "varying dynamics".

We thank the reviewer for their insightful comment and the opportunity to clarify our manuscript. In this case, we are indeed referring specifically to the relative movement between neurons and electrodes over time, which leads to variations in spike shapes—what is commonly described as "drifting" in the spike-sorting literature.

This idea has now been explicitly incorporated on the second paragraph of "*UMAP-based spike sorting achieves superior and robust performance*" in Results as:

"UMAP-based F1 score is also remarkably robust to more complex and challenging perturbations on the recorded waveforms, like drifting, caused by relative movement between neurons and electrodes, and bursting, which involves high-frequency firing of action potentials (Fig. S3C, left and right)."

We have also clarified this definition in Methods, second paragraph of the subsection "*Comparative analysis of different databases*":

"*To test the effectiveness of our method in complex scenarios, we used synthetic datasets simulating electrode drifting and bursty neuronal activity. Electrode drifting was modeled by progressively and linearly decreasing the amplitude of one spike class over time, from a value of 1.0 at the beginning of the recording to 0.3 at the end. Bursty activity was simulated using sequences of consecutive spikes with decaying amplitudes (e.g., 1.0, 0.7, and 0.5), in which action potentials were separated by an average of 3 ms (SD = 1, range: 1–5 ms) (16).*"

18. Methods: How were these bursting and "drifting" simulations constructed? For bursting do you use a model of amplitude modulation vs spike index (as in https://pubmed.ncbi.nlm.nih.gov/32648042/)? What about drifting dynamics? What does it mean, and how is it implemented?

We thank the reviewer for this suggestion, which has improved the organization of our manuscript. In addition to our response to inquiry #17, we have added a new subsection

in the Methods, titled "*Synthetic data simulations*" This section now explicitly details the simulation and application of noise, low-firing-rate ("silent") neurons, drifting, and bursting:

"***Synthetic data simulations***

We utilized the synthetic dataset provided by Quian Quiroga et al. (16) to evaluate sorting performance under controlled conditions (Figs. 2, S3).

Noise and low firing rates: *Background noise (η) is defined as the standard deviation of the background noise relative to the amplitude of the spike waveforms (16). Following the methodology of (16), this noise is modeled as structured (colored) noise, generated by summing spike waveforms from non-sorted background unit at random times. To simulate low firing rates (silent neurons), we randomly subsampled one of the ground truth clusters.*

Bursting activity (amplitude variation): *To simulate the amplitude variations often observed during bursting activity (e.g., amplitude decrease during high-frequency firing), we modulated the amplitude of the spike waveforms within a cluster. The amplitude of the spikes was varied by multiplying the template waveform by a factor drawn from a uniform distribution ranging from 0.5 (50% reduction) to 1.0 (original amplitude).*

Waveform shape variation (Drifting simulation): *To simulate gradual changes in spike shape over time (often caused by electrode drift), we implemented a morphing procedure between two distinct ground truth templates (T1 and T2). The shape of spike k in the sequence was generated as a linear interpolation: $S_k = (1 - \alpha_k)T1 + \alpha_k T2$. The interpolation factor α_k varied linearly from 0 to 1 across the sequence of spikes, simulating a gradual transition from T1 to T2 over the duration of the recording.*"

19: Figure 3: What are the red spike trains?

We appreciate the opportunity to clarify this. In the raster plots for Figures 3, S4, and S5, spike trains from correct trials are shown in black, while those from incorrect trials are shown in red. The corresponding figure captions have been revised to make this explicit.

On Fig. 3:

"*(C) Top: Raster plot of a misclassified multi-unit in the DPC, aligned to the end of (t=0 = start of the delay). Gray rectangles indicate Int1 backpropagated from t=0. Black ticks indicate spikes during correct trials; red ticks indicate spikes during error trials.*"

On Fig. S4:

"(C) Raster (top) and firing rate (middle) of a multi-unit artificially created by pooling two neurons when using PCA-based sorting. Gray shows the stimulus period; black ticks indicate spikes during correct trials; red ticks indicate spikes during error trials."

On Fig. S5:

"(C) Raster plots (top) and firing rate (bottom) for a multi-unit formed by merging two neurons under PCA-based sorting. Trials are aligned to stimulus onset. Blue shading marks stimulus-present trials; gray shading marks stimulus-absent trials. Black ticks indicate spikes during correct trials; red ticks indicate spikes during error trials."

20: Figure 6B: The spike trains of different colors are hard to understand and interpret. How can spike trains represent the "percentage of cluster spikes"?

We thank the reviewer for this valuable feedback. We have revised the manuscript to explicitly define the color-coding scheme used in the raster plots (e.g., Fig. 6B). We now clarify that each plot's background is horizontally split: the color tone of the top half represents the cluster's **Precision**, while the tone of the bottom half represents its **Recall**, with darker shades indicating higher values. This detailed explanation has been added to the second paragraph of the subsection "*Comparing UMAP-based and Feature-based spike sorting in multi-electrode recordings*" in Results:

"*Observe that each raster plot is chromatically split in two halves, distinguished by different blue saturation levels, along the horizontal axis of the plot. The two saturation levels represent the sorting performance of the raster spike cluster with respect to two different metrics, namely, Precision (top half) and Recall (bottom half). Hence, only the raster plots of spike clusters with a high F1 score are drawn in a roughly uniform highly saturated blue tone.*"

And also, in Legend of Fig. 6:

"*(B) Raster plots for GT clusters found by UMAP on each extracellular electrode, alongside the GT neuron's raster. Each raster line is horizontally split; the color saturation of the top half represents the Precision (percentage of cluster spikes that come from the GT), and the color saturation of the bottom half indicates the Recall (percentage of GT spikes captured by that cluster) (see color bar in G).*"

21: Figure S2: letter D is missing

Thank you for pointing this out. We have corrected the figure and added the missing label "D" in the revised version of Figure S2.

Responses to Reviewer #2

Reviewer #2: Summary and impression

In this work, the authors explore non-linear dimensionality reduction methods (specifically, UMAP) for the identification of single units on a single channel during extracellular electrophysiology. They compare this approach with two others that have been used in the past: PCA and wavelet decompositions and clustering methods like a GMM or K-means. They show that not only does UMAP equipped with HDBSCAN (a hierarchical clustering algorithm) better isolate the spikes arising from ground truth single units, they also are able to detect units with very low spike rates; this is something that traditional (density-based) methods fail to cluster. Furthermore, they demonstrate that erroneous isolation of units can confound downstream analyses that depend on assumptions of single unit isolation such as noise correlations and functional cell typing. This paper would be of interest to anyone building spike sorting tools.

Overall, I found the work to make an important and unifying contribution to the spike sorting literature. Specifically, while the utility of non-linear methods has been shown in a scattered way throughout the literature†, it has not been directly shown previously for cortical spikes obtained via threshold crossing and rigorously compared to extant approaches. While not a spike sorting tool, this research provides an important empirical result that I believe everyone working on spike sorting should be aware of.

†P-sort for cerebellum [Sedaghat-Nejad et al., 2021]; WaveMAP for cell type classification [Lee et al. 2023]; unnamed t-SNE method for cell type classification [Jia et al. 2019]; and nearest-neighbor graphs for template-based spike sorting [Pachitariu et al., 2024]

We sincerely thank the reviewer for the positive evaluation of our work and the thoughtful, detailed feedback. We appreciate the recognition of our study as an "important and unifying contribution" and are pleased that the novelty and significance of applying UMAP to threshold-based spike sorting were clear. We have addressed all

the major and minor comments, which has helped improve the manuscript's accuracy and context.

Major Comments

I don't have any major questions or flaws in the work, but I do think that there are a few clarifications/factual inaccuracies that should be included/amended. I view all the numbered major comments as being essential to at least address.

1: This work only is relevant for threshold-based methods. Non-linear methods with hierarchical clustering have also been shown to be highly effective for spike sorting on template-based approaches (Kilosort4). Please be more explicit about this as this result for template-based methods has technically been shown. However, having personally performed threshold- and template-based spike sorting, I believe the two settings are sufficiently different that the novelty of this approach is not in question.

2: Furthermore, Kilosort4 provides an empirical result whereas this work also provides an answer as to "why" non-linear methods are generally superior. Still, I do believe that Pachitariu et al. deserves some mention for their non-linear approach even if they themselves did not make large mention of this change (from density-based methods) in their own work.

We thank the reviewer for emphasizing the need to clarify the scope of our work and properly contextualize it within the landscape of contemporary spike sorting methods, particularly Kilosort4 (KS4). We agree that the distinction between threshold-based and template-based approaches is important.
We have revised the Introduction to explicitly state that our focus is on threshold-based sorting and to acknowledge the success of nonlinear graph-based clustering in template-matching methods like KS4 (Pachitariu et al.).

The revised text (Introduction, Page 3, Paragraph 3) now reads:

> *" The dimensionality reduction of spiking data in traditional threshold-based sorting pipelines is critical, and is typically based on linear methods, ad hoc nonlinear metrics, or deep-learning methods. Template-matching methods like Kilosort4 (22) have also recently demonstrated high performance by incorporating non-linear graph-based clustering (e.g., on nearest-neighbor graphs). We do not directly compare to such methods because our focus here is on improving basic threshold-based spike sorting pipelines, i.e., in a way that is agnostic to specific characteristics of spike waveform. Within this context, we show that the Uniform Manifold Approximation and Projection (UMAP)*

algorithm for unsupervised nonlinear dimensionality reduction drastically increases sorting performance."

3: On the previous point, there are several factual inaccuracies that I believe should be fixed. "Reducing the dimensionality of the processed data is mandatory to apply the clustering algorithms responsible for detecting, isolating and sorting the recorded putative neurons". Technically, this is not "mandatory", but it is however the predominant methodology. I think a phrase such as "near universally applied" or something would be better because it does not imply there is some hard requirement. Factually untrue for spike sorting algorithms writ large because Kilosort4 does also apply clustering in high dimensions as it uses a hierarchical clustering algorithm on the nearest neighbor graph.

We appreciate this correction. We acknowledge that dimensionality reduction is not strictly mandatory, especially given recent advances in high-dimensional graph clustering. We have revised the manuscript to soften this claim.
In the Abstract (Page 2), we changed "*mandatory*" to "*a near-universal practice*."

4: What follows is more of a "statement rather than a question" that I don't expect the authors to incorporate in any way, it's just to make clear my perspective in case it is useful. To my knowledge, nothing fundamentally prevents clustering in ambient space. The good reason for why it is not done is that most methods use metrics inherited from the ambient space (Euclidean distance for example) to establish clusters and these metrics fail to elicit differences (and thus, don't yield meaningful clusters) because of concentration of measure in high-d. This is not just my perspective, it has been explicated by Leland McInnes himself (https://youtu.be/nq6iPZVUxZU?si=_wf554ZnWlYUUm3y&t=674). Graph clustering on a nearest-neighbor graph in high-dimensions (as KS4 does) tends to work very well. The reason why HDBSCAN on a low-d UMAP embedding also works very well is that UMAP too constructs a graph that is projected down into low-d in such a way as to optimize cluster structure over ambient global distance preservation whereas other methods like PCA do not (they preserve global distances). "In all the aforementioned methods, the critical step . . .": So Kilosort4 is cited as one of the "aforementioned methods" but I would not describe it as being "ad-hoc nonlinear". It is no more ad hoc than the approach espoused here by the authors themselves. "To the best of the authors' knowledge, all existing . . .": Well, KS4 and P-sort neither use "expert-defined, nonlinear projections". Also, there is a UMAP visualizer plugin for Phy2 so technically, that is another method.

We thank the reviewer for this insightful perspective on high-dimensional data analysis and for pointing out the mischaracterizations in our text.

We have incorporated the reviewer's explanation regarding the "curse of dimensionality" and "concentration of measure" into the Results section (Page 5, Paragraph 3) to provide a more accurate technical description:

> *"However, clustering in high-dimensional spaces is usually ineffective due to the "curse of dimensionality" (38). This phenomenon leads to the concentration of measure, where standard metrics (e.g., Euclidean distance) fail to reliably distinguish between data points, resulting in unreliable clustering."*

We also updated the description of UMAP to highlight its graph-based nature (Page 6) and corrected the generalizations about existing methods, acknowledging other uses of UMAP (Phy2, P-sort) and ensuring our claims specify "threshold-based" methods where appropriate.

5: *An important point that is elided is that the crucial n_neighbors parameter has a large effect on the size and appearance of embeddings. In my experience, defaults tend to work well for most applications, but this is frequently a point of contention levied by detractors of non-linear approaches. It should be provided some sense of what range of n_neighbor values to use and how HDBSCAN clusters are affected by it. I believe that there is some explanation of this in the "Feature extraction" section of the Methods, but I do not remember this being mentioned in the main text.*

This is an important practical consideration. While UMAP embeddings can visually change with n_neighbors, we found the subsequent clustering by HDBSCAN to be remarkably robust. We have added a new paragraph to the Discussion (Page 19, Paragraph 6) to address the sensitivity to this parameter:

> *" A practical consideration when implementing UMAP is the selection of hyperparameters, most notably the number of neighbors (n_neighbors), which influences the balance between preserving local versus global dataset structures. While this parameter can affect the appearance of the embedding, we found that the sorting results (the clusters identified by HDBSCAN) were robust across a wide range of values. In our analyses, we simply utilized the default UMAP parameters (and in particular n_neighbors=15; see Methods), achieving high performance without extensive tuning. This insensitivity to parameter choice, combined with HDBSCAN's automatic determination of cluster numbers, underscores the suitability of this pipeline for fully unsupervised, high-throughput analysis."*

Minor Comments

1: "Finally, powerful toolkits like KiloSort": the "S" in Kilosort should not be capitalized.

Corrected to "Kilosort" throughout the manuscript.

2: "Projection (P), in which the fuzzy simplicial complex is optimally embedded. . .": So technically an optimization procedure is followed (force-directed graph layout) but this is not "optimal" in any real sense. I would advise just removing the "optimally" from this sentence.

We agree and have removed the word "optimally" (Results, Page 6).

3: Figure 2E: in the legend there is "fr" listed—is this fraction of total firing rate? Some measure of the fraction of false positive spikes? This should be explained in the caption.

We thank the reviewer for the acute observation. We have now clarified this issue in Fig.2' caption, panel (E):

> ***"(E)*** *UMAP-based sorting also proves robust against noise perturbations at various spike dilution levels (dark trace; firing rate [fr] at 100% vs. light blue trace; fr diluted at 40%). Even under increased noise and cluster dilution, it preserves high F1 scores."*

4: "Because noise is a critical factor . . .": What type of noise is this? Gaussian noise applied independently at each time point?

We have clarified the nature of the noise used in the simulations in the Methods section ("*Synthetic data simulations*"). Following the methodology of the benchmark dataset (16), the noise is structured (colored) noise:

> *"Following the methodology of (16), this noise is modeled as structured (colored) noise, generated by summing spike waveforms from non-sorted background units at random times."*

5: "while the (i+1)-th line, i = 1, ..., 4, . . .": The index should end at "3" and not "4" since it's i+1 and there are only four total units shown.

We thank the reviewer for their attention to detail. In Figure 6 (B, I, L), there are five lines shown: the 1st line is the Ground Truth (GT), followed by the four extracellular electrodes (E4-E1) from top (E4) to bottom (E1). The indexing i=1 to 4 correctly describes these four electrodes (Line 5=E1, Line 4=E2, Line 3=E3, Line 2=E4). We have now made the corresponding changes to improve the clarity of this indexing and order.

6: "In spite of this, the utilization of the six most considerable coefficients (illustrated in Figs. 6I and 6J) . . .": I'm not sure how Figs. 6I and 6J make this point? Are the correct figures cited here?

We understand the confusion. Figures 6I and 6J show the *results* of the clustering when using 6 Wavelet components. We have revised the text to clarify this (Results, Page 16):

> *"...the utilization of the six most considerable coefficients enhances the separation process, leading to the improved clustering results shown in Figs. 6I and 6J; however..."*

7: Relative to other contemporary spike sorting approaches with UMAP, like WaveMAP": WaveMAP is not a spike sorting algorithm.

The reviewer is correct that WaveMAP is primarily for cell type classification. We have corrected this categorization in the Discussion (Page 19).

8: I'm not sure if this is a result of prior rounds of review of figure size/number limitations but I found that the supplementary figures so frequently referred to (and used to make important points about results) as to warrant them being main figures. I'm not strongly for or against the restructuring of this but I found it extremely hard to read as is.

We appreciate this feedback regarding the difficulty of reading the paper while frequently referring to supplementary figures. We aimed to maintain a concise main text due to potential journal limitations, but we are happy to reorganize the figures if the editor permits. Furthermore, since this is the first round of revision and all reviewers have made important contributions to the clarity of the manuscript, as well as to the order and **aesthetic** of the figures, we are **confident** that the revised manuscript is now more ordered, concise, and coherent.
