# Supplementary material for: Efficient and reliable spike sorting from neural recordings with UMAP-based unsupervised nonlinear dimensionality reduction
Source: PLoS Biol. 2025 Nov 24;23(11):e3003527. doi: 10.1371/journal.pbio.3003527 (PMC12671831; doi:10.1371/journal.pbio.3003527)
Supplement: Attachment — Submitted filename: Reply to reviewers 2nd - Suarez-Barrera et al.pdf [file pbio.3003527.s012.pdf]

Responses to Reviewer #1

Reviewer #1 (Alessio Paolo Buccino signed his report): The authors addressed all my issues.

I have just one minor comment left, regarding the clarification on "number of session": "*For example, assuming multiple acute sessions per day across a week—resulting in, say, 5 sessions per electrode per week—the use of a 64-electrode multiarray generates approximately 320 (i.e., 64 electrodes recordings times 5 sessions) independent extracellular activity datasets, each containing a variable and unknown number of putative neurons, as a function of largely uncontrolled session-specific conditions.*"
I think that stating that each electrode is independent of each other is wrong. The authors now also incorporated a multi-electrode version and neurons appear on more that one electrode. Honestly, I would just remove the entire sentence: we all agree that with the increasing number of channels manual curation becomes unsuitable.

We agree with Dr. Buccino's assessment. For the sake of clarity, we have removed the confusing sentence from the manuscript. We deeply thank Dr. Buccino for their insightful and constructive feedback throughout the revision process. Their comments have helped us significantly improve the quality and clarity of our manuscript.

Responses to Reviewer #2

Reviewer #2: Overall, I am highly satisfied with the manuscript in both its structure and analyses and believe it will make a high-impact contribution to the field of electrophysiology. It crystallizes the notion that nonlinear methods are superior for clustering which is an essential operation in spike sorting. All of my substantive concerns have been fully addressed and I look forward to seeing this work in print upon which I will immediately it share with my colleagues.

I only had a few very minor edits to make but none are materially important. They only pertain to copyediting and terminology consistency issues especially in the Introduction which could use a closer read. I assume that these will all have been caught anyways in the proofing process but here they are anyways.

- *"To ensure efficient clustering of spiking waveforms, a necessary step in most spike sorting pipeline ...": pipeline should be plural.*
- *"Template-matching methods like Kilosort4 (21, 22) have also recently demonstrated high performance by incorporating non-linear ...": non-linear*

should not be hyphenated for consistency (this also occurs a few other times throughout.

- *"UMAP algorithm (23, 24)": Missing the definite article "The" at the start of this sentence.*
- *"and in efficiently identifying and classifying cell types (26-29). Besides, ...": Usage of the word "besides" is too colloquial in language in my opinion.*
- *"properties of UMAP-based dimensionality reduction make it a perfect candidate ...": Again, the use of "perfect candidate" is probably too colloquial. Consider softening the claim here.*
- *"By reducing data dimension, UMAP automatically identifies nonlinear geometric structures that serve as automatically-discovered geometric (nonlinear)": Nonlinear here is stated twice in the same sentence.*
- *"Moreover, UMAP's robustness to data point density ensures that sparsely represented spike waveforms—such as those from neurons with low firing rates—can efficiently be identified and separated from densely represented waveforms originating from neurons with high firing rates.": I know what is meant here but the phrase "robustness to data point density" is ambiguous for a first-time reader and for its usage for the first time in the text. Consider something more specific such as "ability to capture waveform shape despite low firing rates" or some other explanation. The latter sentence "The proposed spike sorting method particularly stands out for its robustness to even large heterogeneities in the recorded neuron firing rates" is a good one.*
- *"HDBSCAN is a clustering algorithm that does not require to pre-specify ...": should end with, "that does not require "the pre-specification of..."*
- *"Although interesting, we do not explore this analogy further here.": It is highly unsatisfying to end the point here. I believe the authors but an uncharitable reader will not (and most readers are uncharitable).*
- *"over which clustering is feasible, dimensionality-reduction...": Dimensionality reduction should not be hyphenated (this also occurs one other time in the text).*
- *"F1 score: The harmonic mean of Precision and Recall.": Precision and recall should not be capitalized here or anywhere else in the text as they are not proper nouns.*
- *"A high F1 score requires both Precision and Recall being high (the Optimal...": Optimal should not be capitalized.*
- *"This capacity not only reduces manual curation but also ensures complete neuron detection, including silent or low-firing units typically omitted.": What is meant by "silent" is defined in the very next sentence and "silent" should be placed in quotation marks as the neurons are not actually silent but just low-firing rate.*
- *"In contrast to methods like t-SNE or ISOMAP...": Isomap should only have the first letter capitalized.*

We deeply thank the reviewer for their insightful feedback. Their comments were invaluable in helping us improve the rigor, quality, coherence, and flow of the manuscript. We have implemented and addressed all suggested changes.
